# Finerenone and Estimated GFR Slope in Type 2 Diabetes and CKD

**DOI:** 10.1016/j.ekir.2025.04.012

**Published:** 2025-04-14

**Authors:** Masayuki Yamanouchi, Yuki Oba, Hisashi Kamido, Masatoshi Yoshimoto, Yusuke Yoshimura, Hisashi Sugimoto, Shigekazu Kurihara, Akinari Sekine, Tatsuya Suwabe, Naoki Sawa, Takehiko Wada, Kengo Furuichi, Takashi Wada, Yoshifumi Ubara

**Affiliations:** 1 Nephrology Center, Toranomon Hospital, Tokyo, Japan; 2 Nephrology Center, Toranomon Hospital Kajigaya, Kanagawa, Japan; 3 Okinaka Memorial Institute for Medical Research, Tokyo, Japan; 4 Department of Nephrology and Rheumatology, Kanazawa University, Ishikawa, Japan; 5 Department of Nephrology, Kanazawa Medical University School of Medicine, Ishikawa, Japan

**Keywords:** chronic kidney disease, eGFR slope, finerenone, nonsteroidal mineralocorticoid receptor (MR) antagonist, real-world study, type 2 diabetes mellitus

## Introduction

Over the past 2 decades, significant advances have been made in managing chronic kidney disease (CKD) in patients with type 2 diabetes (T2D), with therapies such as renin-angiotensin system (RAS) inhibitors and sodium-glucose cotransporter 2 (SGLT2) inhibitors proving effective in slowing CKD progression.^1^ However, many patients continue to lose kidney function and develop end-stage kidney disease, highlighting an unmet need for treatments that can further slow CKD progression.

Recently, finerenone, a nonsteroidal mineralocorticoid receptor (MR) antagonist, has been shown in clinical trials to slow CKD progression in patients with T2D when combined with RAS inhibitors.^2^^,^^3^ It selectively blocks MR overactivation, which drives inflammation and fibrosis—key contributors to CKD progression—while maintaining serum potassium concentration within an acceptable range, a known concern with steroidal MR antagonists.^4^

Although clinical trials have provided robust evidence for finerenone’s efficacy, its real-world effectiveness in slowing CKD progression remains less well-defined, particularly in patients with a broader range of kidney function and albuminuria. This study evaluates the impact of finerenone on estimated glomerular filtration rate (eGFR) slope improvement in a real-world setting. Detailed methods are described in the Supplementary Methods.

## Results

### Baseline Characteristics

A total of 106 patients who met the inclusion criteria were included in the analysis (the study flow and selection are presented in the Supplementary Figure S1). Baseline characteristics are summarized in Supplementary Table S1. The mean age was 70.8 years (SD: 10.4), with 79.3% male. The median eGFR was 38.9 ml/min per 1.73 m^2^ (interquartile range: 30.1–50.9), and the majority of patients (53.8%) were classified as G3b or lower. The median urine albumin-to-creatinine ratio was 265 mg/g (interquartile range: 43–1578), with 49.1% categorized as A3. Most patients were receiving kidney-protective medications as follows: 82.1% were on RAS inhibitors, 88.7% on SGLT2 inhibitors, and 25.5% on glucagon-like peptide-1 receptor agonists. The median follow-up period after finerenone initiation was 16 months (interquartile range: 14–18).

### Longitudinal Changes in eGFR Before and After Finerenone Initiation

**Figure 1:**
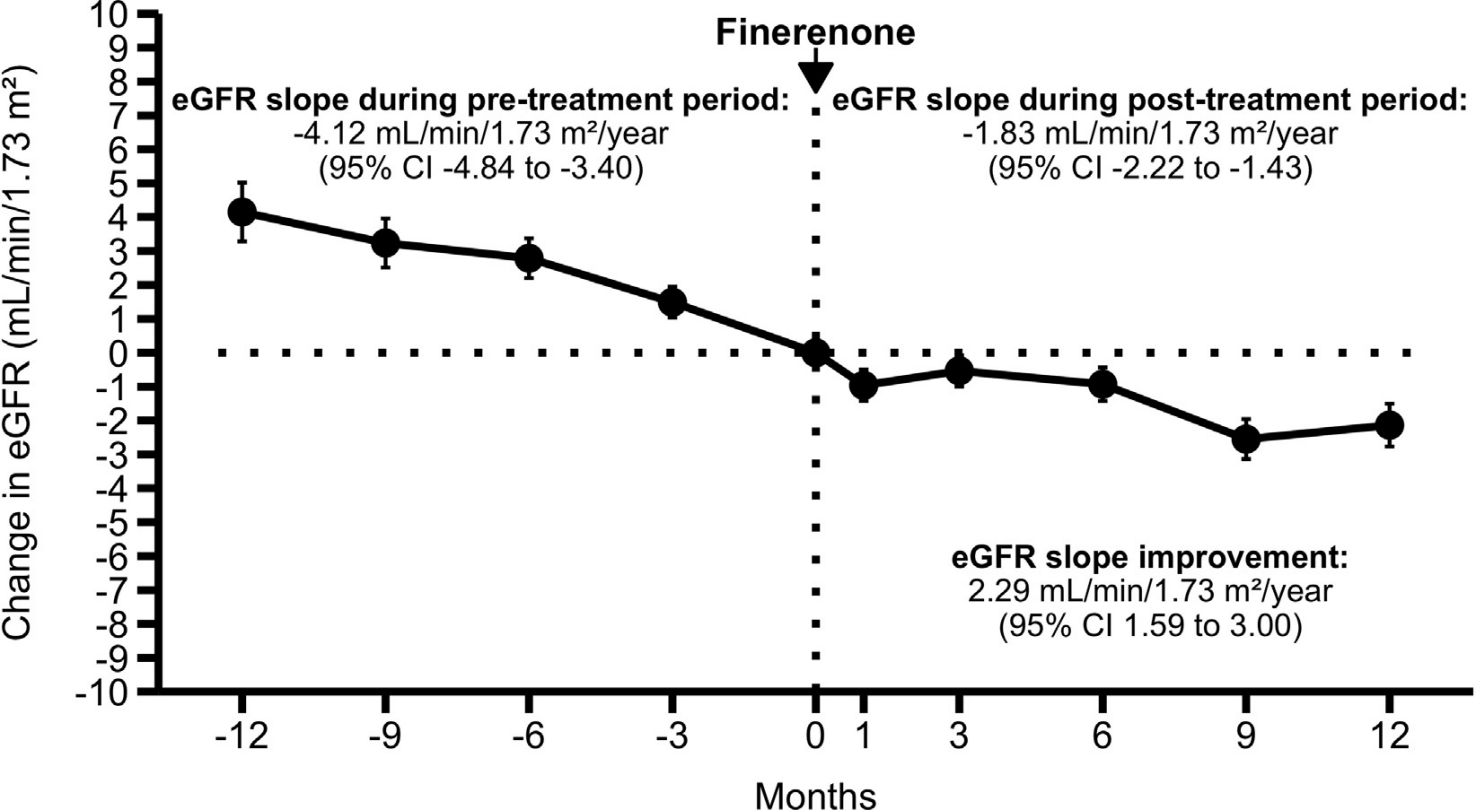
Longitudinal changes in eGFR before and after finerenone initiation. This figure illustrates the longitudinal changes in mean eGFR (± SE) before and after finerenone initiation, with the mean eGFR at month 0 centered to 0. The eGFR slope during the posttreatment period was calculated from 1 month after finerenone initiation to exclude the acute drop in eGFR observed during the first month. Following an acute drop of 0.96 ml/min per 1.73 m^2^ (95% CI: 0.04–1.88), equivalent to a 2.3% decline (95% CI: 0.1–4.5), the eGFR slope improved from −4.12 ml/min per 1.73 m^2^/yr (95% CI: −4.84 to −3.40) to −1.83 ml/min per 1.73 m^2^/yr (95% CI: −2.22 to −1.43). The improvement in eGFR slope was 2.29 ml/min per 1.73 m^2^/yr (95% CI: 1.59–3.00, *P* < 0.001). Linear mixed-effects models for repeated measures were used to estimate these slopes, with additional details provided in the Supplementary Methods section. CI, confidence interval; eGFR, estimated glomerular filtration rate; SE, standard error.

In Figure 1, we illustrate the longitudinal changes in eGFR before and after finerenone initiation, with the mean eGFR at month 0 centered to 0. Finerenone resulted in an acute drop in eGFR of 0.96 ml/min per 1.73 m^2^ (95% confidence interval [CI]: 0.04–1.88), equivalent to a 2.3% decline (95% CI: 0.1–4.5), during the first month. Following this drop, the eGFR slope improved from −4.12 ml/min per 1.73 m^2^/yr (95% CI: −4.84 to −3.40) to −1.83 ml/min per 1.73 m^2^/yr (95% CI: −2.22 to −1.43), representing a difference of 2.29 ml/min per 1.73 m^2^/yr (95% CI: 1.59–3.00, *P* < 0.001). For reference, the same data without baseline centering is provided in Supplementary Figure S2.

### Subgroup Analysis by Baseline eGFR and Albuminuria Categories

**Figure 2:**
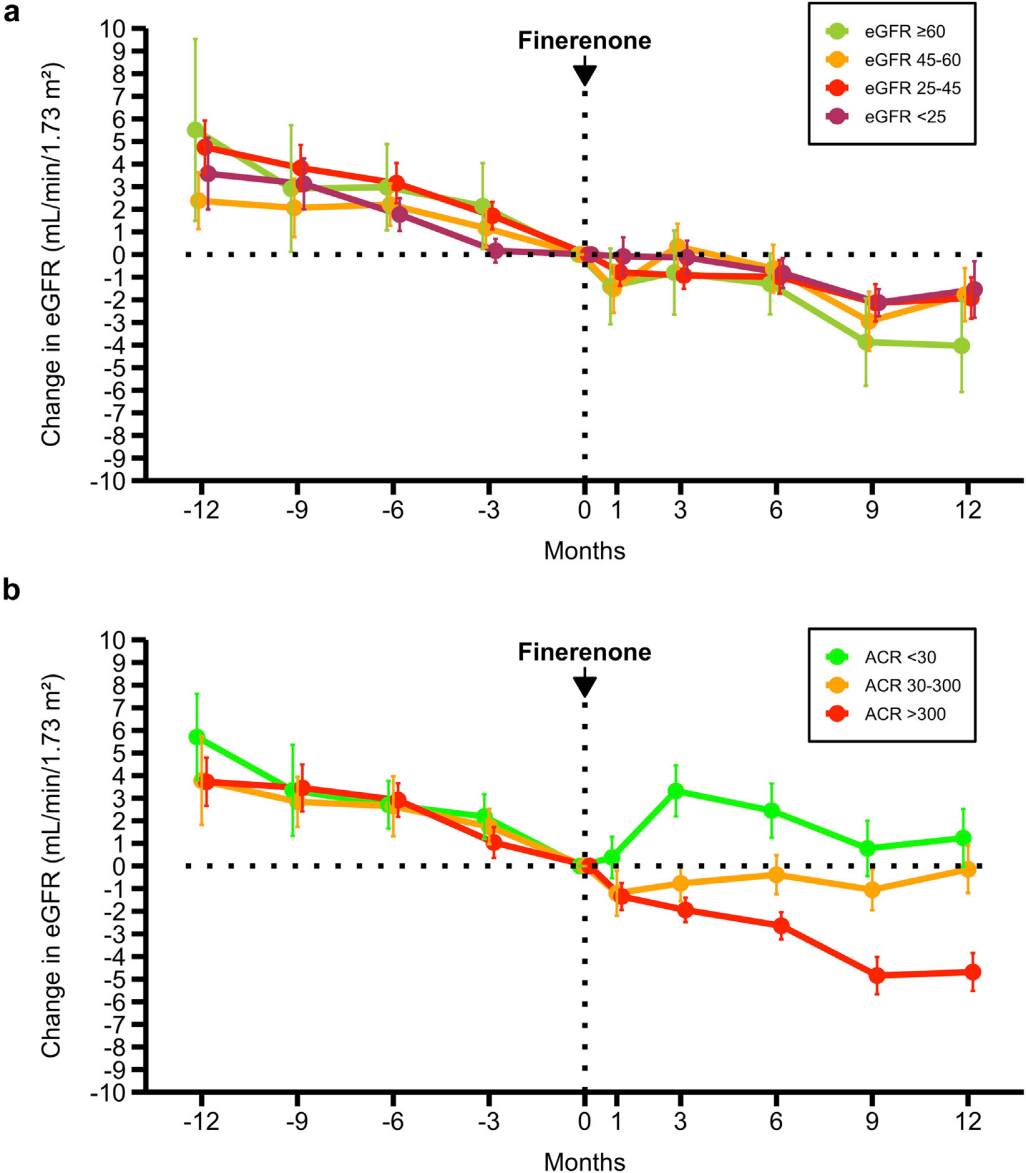
Longitudinal changes in eGFR before and after finerenone initiation by baseline eGFR and albuminuria categories. (a) Longitudinal changes in mean eGFR (± SE) before and after finerenone initiation, stratified by baseline eGFR category. (b) Longitudinal changes in mean eGFR (± SE) before and after finerenone initiation, stratified by baseline albuminuria category. Finerenone improved the eGFR slope regardless of baseline eGFR or albuminuria category. ACR, urine albumin-to-creatinine ratio; eGFR, estimated glomerular filtration rate.

Finerenone improved the eGFR slope across all baseline eGFR and albuminuria categories (Figure 2 and Supplementary Table S2). The same data without baseline centering is provided in Supplementary Figure S3.

### Supplementary Results

Supplementary results, including the relationship between the magnitude of the acute drop in eGFR and eGFR slope improvement (Supplementary Table S3 and Figure S4), longitudinal changes in albuminuria (Supplementary Figure S5), serum potassium concentration (Supplementary Figure S6), and blood pressure (Supplementary Figure S7), are provided in the Supplementary Results. The effects of finerenone on eGFR slope according to the concomitant use of RAS inhibitors, SGLT2 inhibitors, and glucagon-like peptide-1 receptor agonists are described in Supplementary Figure S9, S10, and S11.

## Discussion

This real-world analysis shows that finerenone improved the eGFR slope in patients with T2D and CKD, regardless of baseline eGFR and albuminuria levels, without notable hyperkalemia.

Whereas improvements in hard kidney failure outcomes, such as progression to end-stage kidney disease, may represent the most meaningful clinical benefit, assessing a surrogate kidney failure outcome—eGFR slope—provides valuable insights. Changes in eGFR slope are strongly associated with kidney failure outcomes and allow data from all study participants to be analyzed.^5^ Given the 12-month study duration, observing hard kidney failure outcomes was not feasible, especially in patients with preserved kidney function or normoalbuminuria. Therefore, eGFR slope was adopted as the primary outcome to better evaluate finerenone's impact on kidney function. The variability in eGFR decline necessitated the use of a linear mixed-effects model, which accounted for variations in eGFR slopes both between and within patients. With this model, we found that finerenone significantly slowed the rate of eGFR decline across all baseline eGFR and albuminuria levels, supporting its utility in preserving kidney function in real-world patients with T2D and CKD.

Steroidal MR antagonists such as spironolactone or eplerenone provide benefits for both the heart and kidneys; however, their widespread use has been linked to increased hyperkalemia.^6^ In contrast, finerenone, a nonsteroidal MR antagonist, offers similar benefits with minimal risk of hyperkalemia.^2^^,^^3^ In this study, serum potassium concentration increased slightly but remained within acceptable limits, with low incidence of hyperkalemia. This low incidence of hyperkalemia-related adverse events aligns with real-world studies that report a similar rate of hyperkalemia (2–3 cases per 100 patients), with no hospitalizations because of hyperkalemia reported.^7^

An acute drop in eGFR observed after initiating drugs such as RAS inhibitors and SGLT2 inhibitors—believed to reflect the correction of glomerular hypertension—has been linked to slower kidney function decline (Supplementary References).^8^ Similarly, in this study, finerenone caused an acute drop in eGFR, which was correlated with eGFR slope improvement, suggesting it may be a marker of kidney protection. Interestingly, a transient increase in eGFR was observed in patients with urine albumin-to-creatinine ratio < 30 mg/g, yet finerenone still improved the eGFR slope in this subgroup. This aligns with findings from the EMPA-REG OUTCOME and CREDENCE trials, where 27% to 30% of patients showed an acute increase in eGFR, although these findings were not specific to patients with urine albumin-to-creatinine ratio < 30 mg/g.^9^ This phenomenon suggests that certain subgroups may respond differently to interventions, possibly because of differences in underlying glomerular hypertension. The fact that eGFR slope improvement was still observed in this subgroup implies that mechanisms in addition to the correction of glomerular hypertension may contribute to finerenone’s beneficial effects, warranting further investigation.

Key strengths of this study include its real-world setting, which included a broader range of kidney function and albuminuria levels compared with clinical trials, making the findings more applicable to routine practice. The analysis of eGFR slope improvement provides new insights into early predictors of treatment response.

However, some limitations should be acknowledged. The cohort was predominantly Japanese, which may limit generalizability. The sample size was relatively small, and the short follow-up period may affect the robustness of the results. Furthermore, clinical trials demonstrated finerenone’s effects in patients receiving the maximum tolerated dose of RAS inhibitors, whereas only 25.5% of our cohort was on maximum-dose RAS inhibitors. In addition, nearly 90% of our cohort was on SGLT2 inhibitors, which were less commonly used in clinical trials. These differences may limit the comparability of our findings with clinical trials and highlight the need for further research to clarify the optimal combination and sequencing of therapies for patients with T2D and CKD.

In conclusion, this real-world analysis provides evidence that finerenone may improve the eGFR slope in patients with T2D and CKD, regardless of baseline eGFR and albuminuria levels, without notable hyperkalemia. The acute drop in eGFR may serve as an early predictor of eGFR slope improvement. These findings support finerenone as a valuable therapeutic option in real-world settings.

## Disclosure

MY reports personal honoraria for lectures by Bayer Pharma. All the other authors declared no competing interests.

## Supplementary Material

Supplementary File (PDF) **Supplementary Methods. Supplementary Results. Supplementary References. Figure S1.** Study flow. **Figure S2.** Longitudinal changes in eGFR before and after finerenone initiation without baseline eGFR centering. **Figure S3.** Longitudinal changes in eGFR before and after finerenone initiation by baseline eGFR and albuminuria categories without baseline eGFR centering. **Figure S4.** eGFR slope improvement by percentage of acute drop in eGFR. **Figure S5.** Longitudinal changes in urine albumin-to-creatinine ratio after finerenone initiation. **Figure S6.** Longitudinal changes in serum potassium concentration after finerenone initiation. **Figure S7.** Longitudinal changes in blood pressure after finerenone initiation. **Figure S8.** Individual eGFR trajectories and mean eGFR trajectories before and after finerenone initiation. **Figure S9.** Longitudinal changes in eGFR before and after finerenone initiation according to concomitant use of RAS inhibitors. **Figure S10.** Longitudinal changes in eGFR before and after finerenone initiation according to concomitant use of SGLT2 inhibitors. **Figure S11.** Longitudinal changes in eGFR before and after finerenone initiation according to concomitant use of glucagon-like peptide-1 receptor agonists. **Table S1.** Baseline characteristics of the study population. **Table S2.** Effect of finerenone on eGFR slope changes stratified by baseline eGFR and urine albumin-to-creatinine ratio categories. **Table S3.** The relationship between acute drop in eGFR and eGFR slope improvement. STROBE Checklist.

## Footnotes

Supplementary File (PDF)

**Supplementary Methods.**

**Supplementary Results.**

**Supplementary References.**
**Figure S1.** Study flow. **Figure S2.** Longitudinal changes in eGFR before and after finerenone initiation without baseline eGFR centering. **Figure S3.** Longitudinal changes in eGFR before and after finerenone initiation by baseline eGFR and albuminuria categories without baseline eGFR centering. **Figure S4.** eGFR slope improvement by percentage of acute drop in eGFR. **Figure S5.** Longitudinal changes in urine albumin-to-creatinine ratio after finerenone initiation. **Figure S6.** Longitudinal changes in serum potassium concentration after finerenone initiation. **Figure S7.** Longitudinal changes in blood pressure after finerenone initiation. **Figure S8.** Individual eGFR trajectories and mean eGFR trajectories before and after finerenone initiation. **Figure S9.** Longitudinal changes in eGFR before and after finerenone initiation according to concomitant use of RAS inhibitors. **Figure S10.** Longitudinal changes in eGFR before and after finerenone initiation according to concomitant use of SGLT2 inhibitors. **Figure S11.** Longitudinal changes in eGFR before and after finerenone initiation according to concomitant use of glucagon-like peptide-1 receptor agonists. **Table S1.** Baseline characteristics of the study population. **Table S2.** Effect of finerenone on eGFR slope changes stratified by baseline eGFR and urine albumin-to-creatinine ratio categories. **Table S3.** The relationship between acute drop in eGFR and eGFR slope improvement. STROBE Checklist.
