# Supplementary material for: Finerenone and Estimated GFR Slope in Type 2 Diabetes and CKD
Source: Kidney Int Rep. 2025 Apr 14;10(7):2461–5. doi: 10.1016/j.ekir.2025.04.012 (PMC12266157; doi:10.1016/j.ekir.2025.04.012)
Supplement: Supplementary File (PDF) — Supplementary Methods. Supplementary Results. Supplementary References. Figure S1. Study flow. Figure S2. Longitudinal changes in eGFR before and after finerenone initiation without baseline eGFR centering. Figure S3. Longitudinal changes in eGFR before and after finerenone initiation by baseline eGFR and albuminuria categories without baseline eGFR centering. Figure S4. eGFR slope improvement by percentage of acute drop in eGFR. Figure S5. Longitudinal changes in urine albumin-to-creatinine ratio after finerenone initiation. Figure S6. Longitudinal changes in serum potassium concentration after finerenone initiation. Figure S7. Longitudinal changes in blood pressure after finerenone initiation. Figure S8. Individual eGFR trajectories and mean eGFR trajectories before and after finerenone initiation. Figure S9. Longitudinal changes in eGFR before and after finerenone initiation according to concomitant use of RAS inhibitors. Figure S10. Longitudinal changes in eGFR before and after finerenone initiation according to concomitant use of SGLT2 inhibitors. Figure S11. Longitudinal changes in eGFR before and after finerenone initiation according to concomitant use of glucagon-like peptide-1 receptor agonists. Table S1. Baseline characteristics of the study population. Table S2. Effect of finerenone on eGFR slope changes stratified by baseline eGFR and urine albumin-to-creatinine ratio categories. Table S3. The relationship between acute drop in eGFR and eGFR slope improvement. STROBE Checklist. [file mmc1.pdf]

SUPPLEMENTARY MATERIAL

Finerenone and Estimated GFR Slope in Type 2 Diabetes and CKD

Masayuki Yamanouchi[1,2,3,4,5], Yuki Oba[2], Hisashi Kamido[2], Masatoshi Yoshimoto[2], Yusuke Yoshimura[2], Hisashi Sugimoto[2], Shigekazu Kurihara[2], Akinari Sekine[1], Tatsuya Suwabe[1,2], Naoki Sawa[1,2], Takehiko Wada[1], Kengo Furuichi[5], Takashi Wada[4], and Yoshifumi Ubara[1,2]

[1] Nephrology Center, Toranomon Hospital, Tokyo, Japan
[2] Nephrology Center, Toranomon Hospital Kajigaya, Kanagawa, Japan
[3] Okinaka Memorial Institute for Medical Research, Tokyo, Japan
[4] Department of Nephrology and Rheumatology, Kanazawa University, Ishikawa, Japan
[5] Department of Nephrology, Kanazawa Medical University School of Medicine, Ishikawa, Japan

Correspondence:
Masayuki Yamanouchi
Nephrology Center, Toranomon Hospital, 2-2-2 Toranomon, Minato-ku, Tokyo 105-8470, Japan
E-mail: m.yamanouchi@toranomon.gr.jp

Supplementary Methods

Study Design

This retrospective, observational study was conducted in a real-world clinical setting, including patients with T2D and CKD. The study was approved by the institutional review boards of Toranomon Hospital (Tokyo, Japan) and Toranomon Hospital Kajigaya (Kanagawa, Japan). It adhered to the Declaration of Helsinki, and patient confidentiality was maintained through data anonymization.

Study Population

The study included patients with T2D and CKD who were regularly followed at Toranomon Hospital and Toranomon Hospital Kajigaya. Patients eligible for inclusion were those aged 18 years or older, diagnosed with CKD stages 1-5 (not on dialysis), and who newly initiated finerenone between July 2022 and July 2023. Additional inclusion criteria required available eGFR and ACR data from at least 12 months before finerenone initiation and a follow-up period of at least 12 months after finerenone initiation. Patients who had previously undergone dialysis or received a kidney transplant were excluded.

Finerenone Dose Titration

Finerenone dosing was initiated and adjusted primarily in accordance with the prescribing information. The starting dose was determined based on baseline eGFR and serum potassium concentration: 10 mg once daily for patients with an eGFR <60

mL/min/1.73 m^2 and 20 mg once daily for those with an eGFR ≥60 mL/min/1.73 m^2, provided the serum concentration was ≤4.8 mmol/L.

Dose titration and adjustments were performed at 1 month, following the protocol below:

- Serum potassium concentration ≤4.8 mmol/L: Maintain 20 mg once daily. For patients on 10 mg once daily, increase the dose to 20 mg once daily if eGFR had not decreased by more than 30% compared to the previous measurement.
- Serum potassium concentration 4.9–5.5 mmol/L: Maintain the current dose.
- Serum potassium concentration >5.5 mmol/L: Withhold finerenone. Treatment was resumed at 10 mg once daily if serume potassium concentration decreased to ≤5.0 mmol/L.

Data Source

Patient profiles and laboratory results, including age, sex, body mass index (BMI), systolic blood pressure (SBP), diastolic blood pressure (DBP), duration of diabetes, hematology, glycated hemoglobin (HbA_{1c}), serum creatinine, total cholesterol, low-density lipoprotein (LDL) cholesterol, high-density lipoprotein (HDL) cholesterol, triglycerides, and urine albumin-to-creatinine ratio (ACR), were retrieved from electronic medical records. Medication usage, including RAS inhibitors, SGLT2 inhibiitors, glucagon-like peptide-1 (GLP-1) receptor agonists, statins, potassium binders, and diuretics, was also documented. Estimated glomerular filtration rate (eGFR) was calculated using the Chronic Kidney Disease Epidemiology Collaboration (CKD-EPI) equation modified by a Japanese coefficient.[S1] Longitudinal eGFR data were obtained from 12 months before finerenone initiation to 12 months after.

Baseline eGFR categories were defined as follows: ≥60 mL/min/1.73 m², 45-59 mL/min/1.73 m², 25-44 mL/min/1.73 m², and <25 mL/min/1.73 m². Albuminuria categories at baseline were classified according to KDIGO guidelines[S2] as follows: A1 (ACR of <30 mg/g), A2 (ACR 30 to 300 mg/g), and A3 (ACR >300 mg/g), correspong to normoalbuminuria, microalbuminuria, and macroalbuminuria, respectively.

Outcome Measures

The primary outcome was the change in eGFR slope during the pre-treatment and post-treatment periods. For the post-treatment eGFR slope, the chronic slope, calculated from 1 month after finerenone initiation to exclude the acute drop in eGFR, was used. Previous studies on the acute drop in eGFR and chronic eGFR slope with finerenone[S3] and SGLT2 inhibitors[S4-S6] have generally defined the acute drop as occurring within 2 weeks to 3 months, although a precise definition is not clearly established. Notably, the CANPIONE study[S6], which followed patients for 12 months after canagliflozin administration—similar to the follow-up period in our study—defined the acute drop period as 1 month. Based on this reference, we defined the chronic slope as starting from 1 month after finerenone initiation to exclude the acute drop in eGFR.

Secondary outcomes included the correlations between the magnitude of the acute drop in eGFR and changes in eGFR slope.

Statistical Analyses

To assess whether a linear relationship between eGFR and time is reasonable for the application of a linear mixed-effects model, we created individual “spaghetti plots” with

overall mean eGFR plots to evaluate whether the eGFR trajectories appear approximately linear before and after finerenone initiation.

To compare the pre-treatment and post-treatment eGFR slopes, we used a linear mixed-effects model with a random intercept and a random slope to account for inter-individual variability in baseline eGFR and rates of eGFR decline. Given that the post-treatment slope was calculated starting from 1 month after finerenone initiation to exclude the acute drop, only data from 12 months to 0 months before initiation and 1 month to 12 months after initiation were included in the analysis.

The model was specified as:

$eGFR_{ij} = \beta_0 + \beta_1 \cdot time_{ij} + \beta_2 \cdot treatment_{ij} + \beta_3 \cdot (time_{ij} \times treatment_{ij}) + u_{0i} + u_{1i} \cdot time_{ij} + \epsilon_{ij}$

where:

- $eGFR_{ij}$ represents the eGFR for patient i at time point j.
- $time_{ij}$ is the time from finerenone initiation in months (centered at 0).
- $treatment_{ij}$ is a binary variable indicating whether the measurement was taken before (0) or after (1) finerenone initiation.
- $time_{ij} \times treatment_{ij}$ represents the interaction term, capturing the difference in eGFR slope before and after finerenone initiation.
- u_{0i} and u_{1i} are patient-specific random effects for the intercept and slope, respectively.
- ϵ_{ij} is the residual error term.

The model was fitted with an unstructured random-effects covariance structure to allow flexibility in modeling inter-individual variations in eGFR trajectories. The coefficient of primary interest was the interaction term (β_3), which represents the difference in eGFR

slope between the pre- and post-treatment periods. A statistically significant β_3 ($P < 0.05$) indicates that finerenone significantly altered the rate of eGFR decline. To adjust for potential confounders, baseline characteristics were included in the model, such as age, sex, diabetes duration, BMI, systolic blood pressure, HbA1c, LDL cholesterol, eGFR, ACR, and the use of RAS inhibitors, SGLT2 inhibitors, and GLP-1 receptor agonists. Subgroup analyses were conducted across different baseline eGFR and albuminuria categories using the same model structure.

For secondary analyses, Pearson's correlation coefficient was used to evaluate the relationship between the magnitude of the acute drop in eGFR and eGFR slope improvement. Additionally, eGFR slope improvement was analyzed across categorical percentages of the acute drop in eGFR (0%, 0-10%, and >10%), as these cutoffs have been commonly used in previous studies investigating SGLT2 inhibitors[S4,S5], despite the lack of a standardized classification.

For supplementary analyses, longitudinal changes in ACR, serum potassium concentration, and blood pressure (SBP and DBP) were assessed to evaluate potential effects of finerenone. Graphical representations were provided to illustrate longitudinal trends for these variables during the follow-up period after finerenone initiation. Additionally, to examine whether the effects of finerenone on eGFR slope differ depending on the concomitant use of RAS inhibitors, SGLT2 inhibitors, and GLP-1 receptor agonists, we performed subgroup analyses stratified by the presence or absence of these medications.

Descriptive statistics were presented as mean and standard deviation (SD), median and interquartile range (IQR), or proportions as appropriate. All statistical

analyses were conducted using Stata version 18.5 (StataCorp LLC, College Station, TX, USA), with a two-sided *P* value <0.05 considered statistically significant.

Supplementary Results

Individual "spaghetti plots" with overall mean eGFR plots before and after finerenone initiation

The individual "spaghetti plots" with overall mean eGFR plots demonstrated that the mean eGFR trajectories appeared approximately linear before and after finerenone initiation. This finding supports the appropriateness of applying a linear mixed-effects model. The plots are provided as Supplemental Figure S8.

Relationships between the magnitude of the acute drop in eGFR and eGFR slope improvement

While slope improvement was observed regardless of the magnitude of the acute drop in eGFR after finerenone initiation, a positive correlation was identified between the acute drop and eGFR slope improvement ($r = 0.43$, $P < 0.001$) (Supplementary Table S3). Supplementary Figure S4 shows the relationship between the categorical percentages of the acute drop in eGFR (<0%, 0-10%, and >10%) and eGFR slope improvement. Among the categories, 19 patients had a <0% drop, 43 patients had a 0-10% drop, and the remaining 44 patients experienced a >10% drop. eGFR slope improvement was observed across all categories of acute drop in eGFR, with patients experiencing a >10% drop demonstrating the most pronounced improvement.

Longitudinal changes in albuminuria, serum potassium concentration, and blood pressure

Supplementary Figure S5 illustrates the longitudinal changes in the least-squares mean ratio to baseline of ACR, showing that finerenone gradually reduced ACR over time, with the most pronounced decrease observed after 6 months. By month 12, ACR had decreased by approximately 21% from baseline, with a least-squares mean ratio to baseline of 0.79 (95% CI 0.61 to 0.96). Supplementary Figure S6 shows the longitudinal changes in serum potassium concentration, which increased slightly after finerenone initiation but remained within the acceptable range during follow-up. Of note, three patients experienced hyperkalemia (serum potassium concentration ≥5.5 mmol/L), which required temporary discontinuation of finerenone. No life-threatening hyperkalemia or hospitalizations due to hyperkalemia occurred during the study period. Supplementary Figure S7 describes longitudinal changes in blood pressure, showing stable mean SBP and DBP throughout the 12-month follow-up period.

Effects of finerenone on eGFR improvement according to concomitant use of other kidney-protective medications

Finerenone improved the eGFR slope regardless of the concomitant use of RAS inhibitors, SGLT2 inhibitors, and GLP-1 receptor agonists (Supplementary Figure S9-S11).

Supplementary References

S1. Horio M, Imai E, Yasuda Y, Watanabe T, Matsuo S. Modification of the CKD epidemiology collaboration (CKD-EPI) equation for Japanese: accuracy and use for population estimates. *Am J Kidney Dis*. 2010;56(1):32-38. doi:10.1053/j.ajkd.2010.02.344

S2. Kidney Disease: Improving Global Outcomes (KDIGO) CKD Work Group. KDIGO 2024 Clinical Practice Guideline for the Evaluation and Management of Chronic Kidney Disease. *Kidney Int*. 2024;105(4S):S117-S314. doi:10.1016/j.kint.2023.10.018

S3. Bakris GL, Ruilope LM, Anker SD, et al. A prespecified exploratory analysis from FIDELITY examined finerenone use and kidney outcomes in patients with chronic kidney disease and type 2 diabetes. *Kidney Int*. 2023;103(1):196-206. doi:10.1016/j.kint.2022.08.040

S4. Kraus BJ, Weir MR, Bakris GL, et al. Characterization and implications of the initial estimated glomerular filtration rate 'dip' upon sodium-glucose cotransporter-2 inhibition with empagliflozin in the EMPA-REG OUTCOME trial. *Kidney Int*. 2021;99(3):750-762. doi:10.1016/j.kint.2020.10.031

S5. Oshima M, Jardine MJ, Agarwal R, et al. Insights from CREDENCE trial indicate an acute drop in estimated glomerular filtration rate during treatment with canagliflozin with implications for clinical practice. *Kidney Int*. 2021;99(4):999-1009. doi:10.1016/j.kint.2020.10.042

S6. Miyamoto S, Heerspink HJL, de Zeeuw D, et al. A randomized, open-label, clinical trial examined the effects of canagliflozin on albuminuria and eGFR decline using an individual pre-intervention eGFR slope. *Kidney Int*. 2024;106(5):972-984. doi:10.1016/j.kint.2024.08.019

Supplementary Figures

Supplementary Figure S1. Study flow.

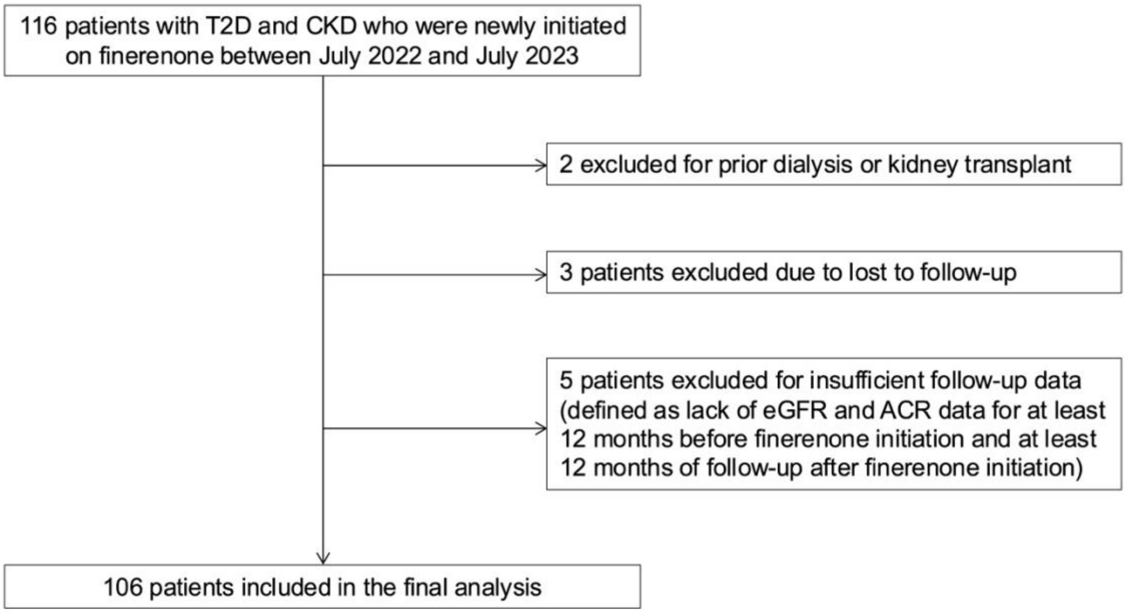

Supplementary Figure S2. Longitudinal changes in eGFR before and after finerenone initiation without baseline eGFR centering.

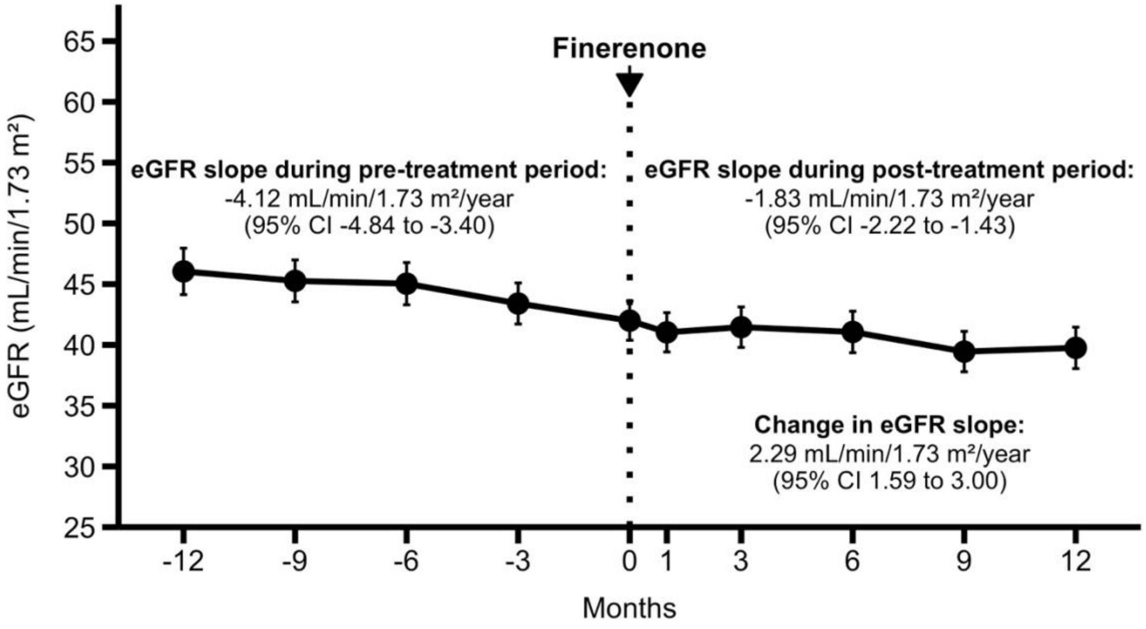

This figure illustrates the longitudinal changes in mean eGFR (±SE) before and after finerenone initiation. The eGFR slope during the post-treatment period was calculated from 1 month after finerenone initiation to exclude the acute drop in eGFR observed during the first month.

Following an acute drop of 0.96 mL/min/1.73 m² (95% CI 0.04 to 1.88), equivalent to a 2.3% decline (95% CI 0.1 to 4.5), the eGFR slope improved from -4.12 mL/min/1.73 m²/year (95% CI -4.84 to -3.40) to -1.83 mL/min/1.73 m²/year (95% CI -2.22 to -1.43). The improvement in eGFR slope was 2.29 mL/min/1.73 m²/year (95% CI 1.59 to 3.00, P <0.001). Linear mixed-effects models for repeated measures were used to estimate these slopes, with additional details provided in the Suppementary Methods section.

eGFR, estimated glomerular filtration rate.

Supplementary Figure S3. Longitudinal changes in eGFR before and after finerenone initiation by baseline eGFR and albuminuria categories without baseline eGFR centering.

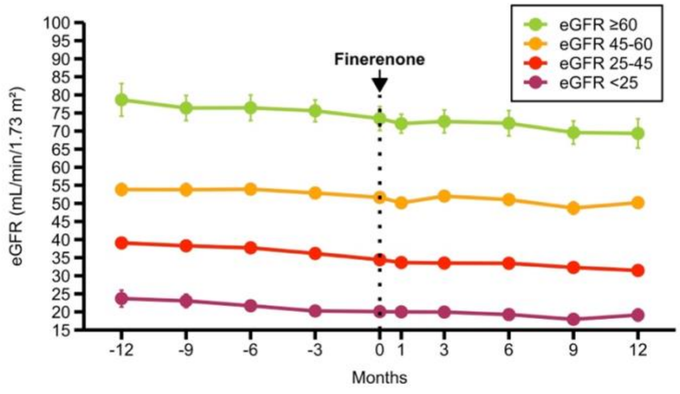

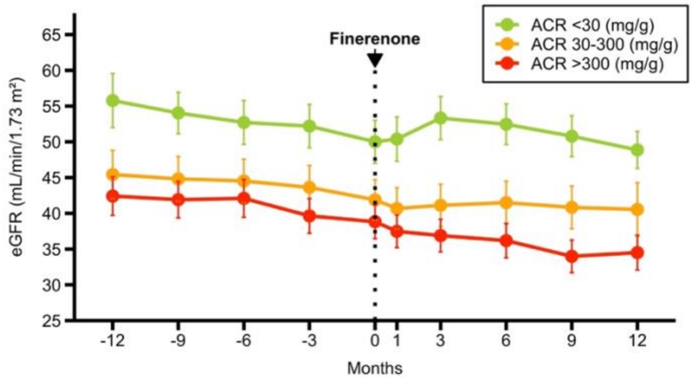

(a) Longitudinal changes in mean eGFR (±SE) before and after finerenone initiation, stratified by baseline eGFR category. (b) Longitudinal changes in mean eGFR (±SE) before and after finerenone initiation, stratified by baseline albuminuria category. Finerenone improved the eGFR slope regardless of baseline eGFR or albuminuria category. ACR, urine albumin-to-creatinine ratio.

Supplementary Figure S4. eGFR slope improvement by percentage of acute drop in eGFR.

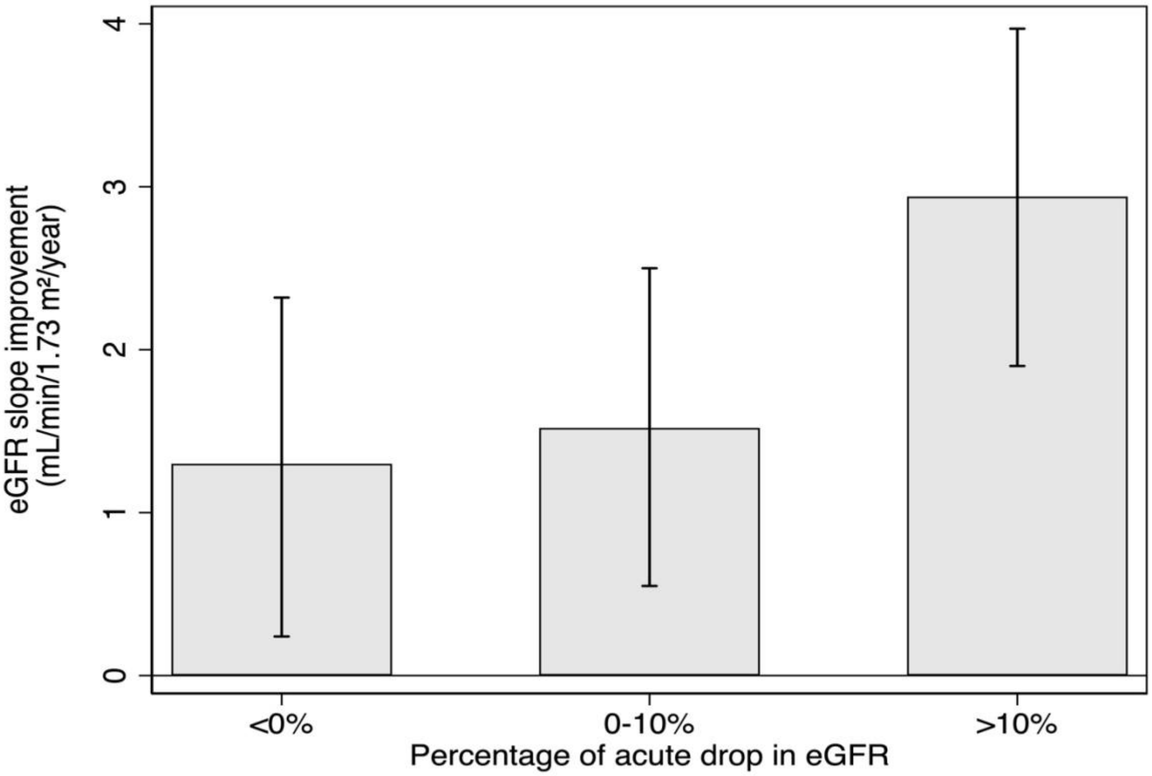

This figure illustrates the relationship between the categorical percentages of the acute drop in eGFR (<0%, 0-10%, and >10%) and eGFR slope improvement. Data represent the mean changes in eGFR slope (mL/min/1.73 m^2/year) with error bars indicating 95% confidence intervals. eGFR improvement was observed across all categories, with the most pronounced improvement seen in patients with a >10% acute drop in eGFR. eGFR, estimated glomerular filtration rate.

Supplementary Figure S5. Longitudinal changes in urine albumin-to-creatinine ratio after finerenone initiation.

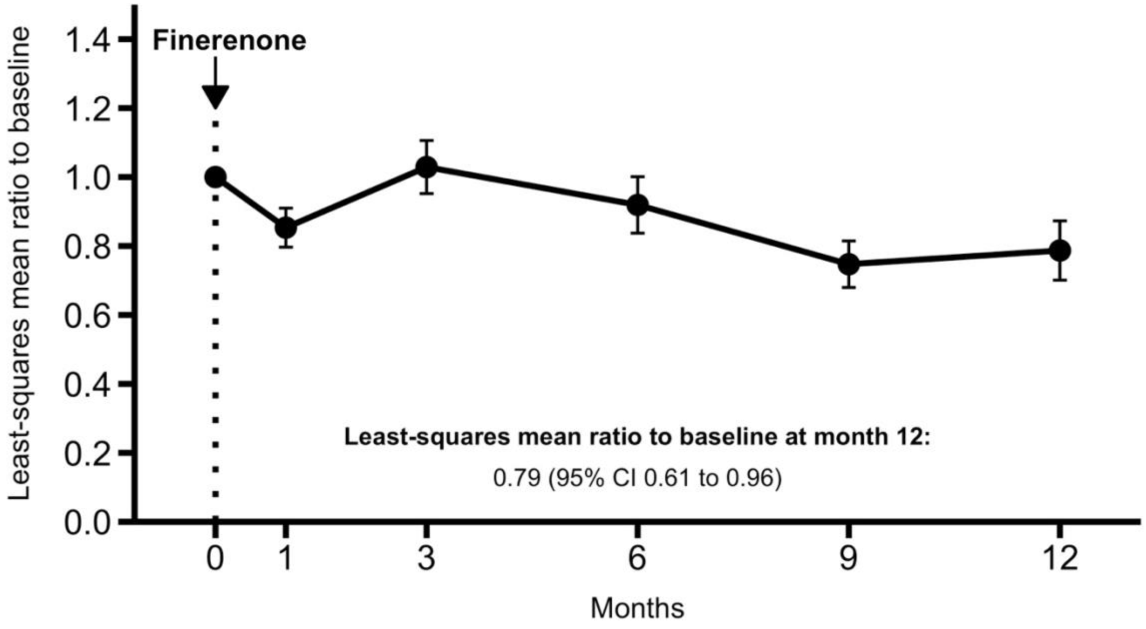

Supplementary Figure S6. Longitudinal changes in serum potassium concentration after finerenone initiation.

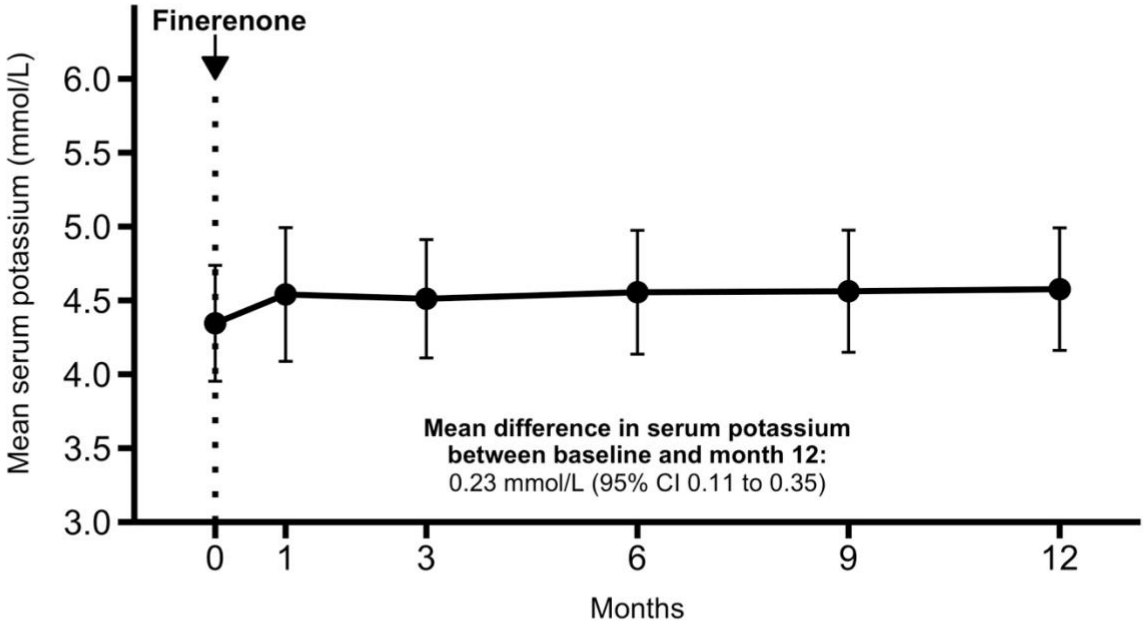

Supplementary Figure S7. Longitudinal changes in blood pressure after finerenone initiation. (a) Longitudinal changes in systolic blood pressure after finerenone initiation. (b) Longitudinal changes in diastolic blood pressure after finerenone initiation.

a

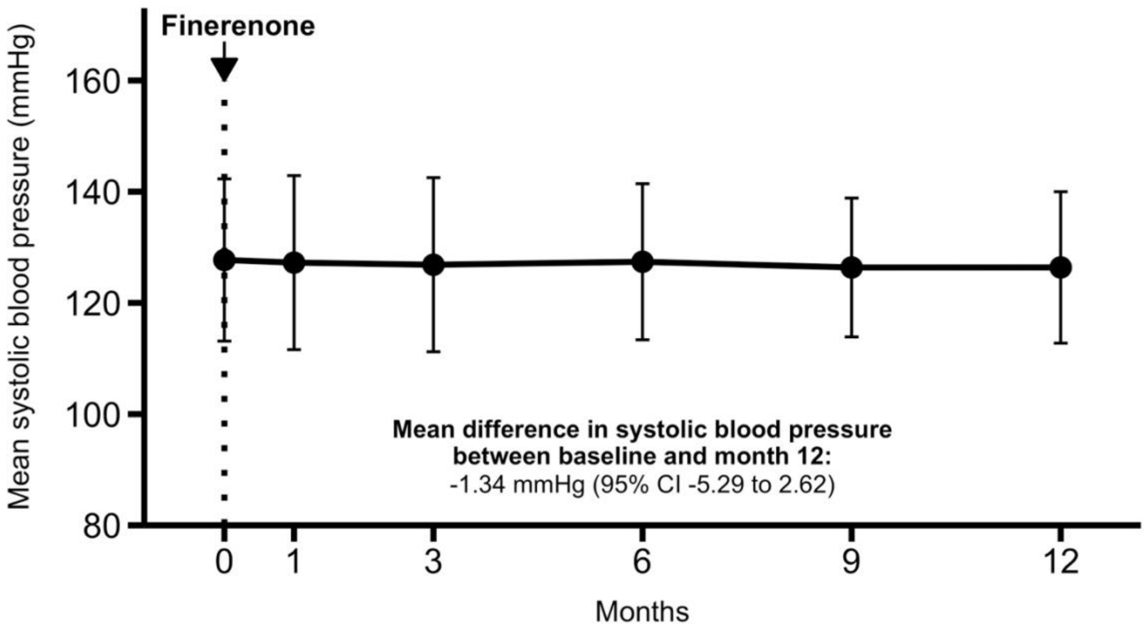

b

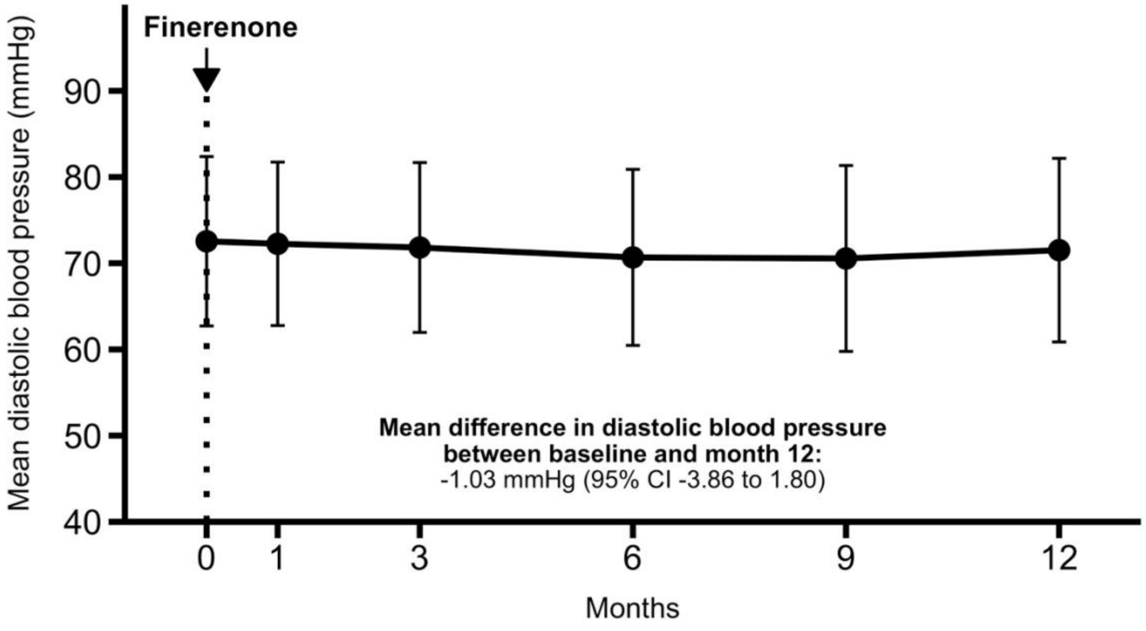

Supplementary Figure S8. Individual eGFR trajectories and mean eGFR trajectories before and after finerenone initiation.

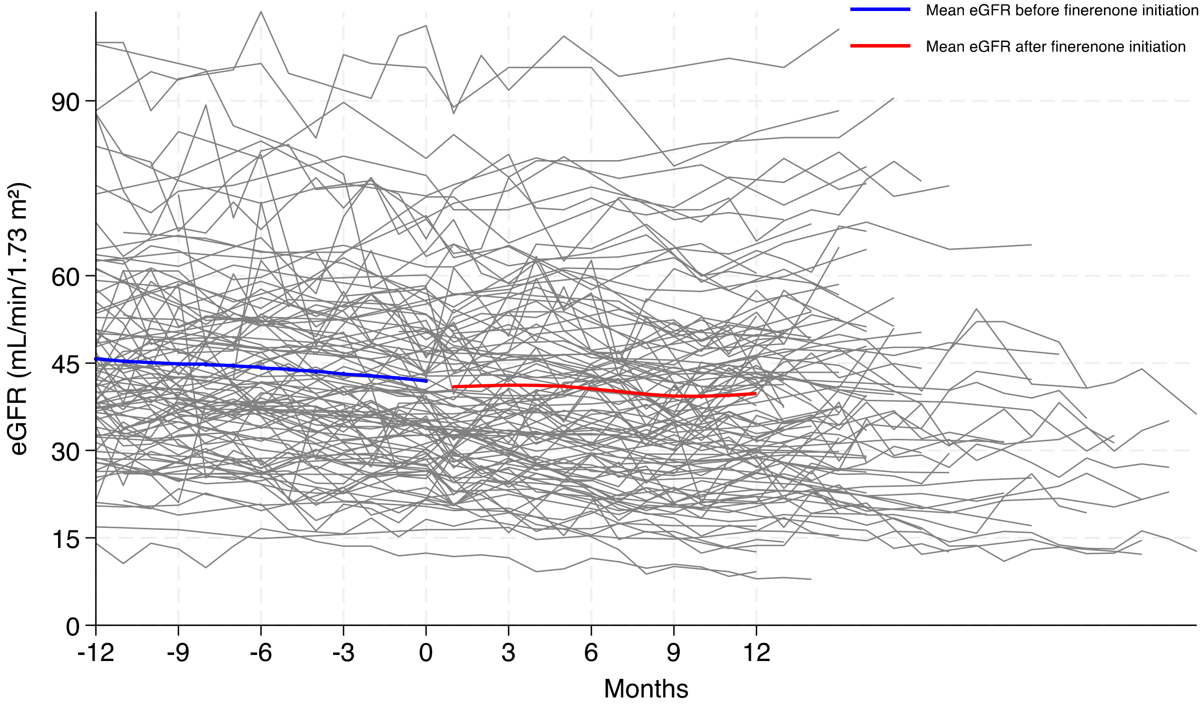

The plot shows individual eGFR trajectories (thin gray lines) of 106 patients from 12 months before to at least 12 months after finerenone initiation. The blue line represents the mean eGFR before finerenone initiation (month -12 to 0), while the red line represents the mean eGFR after finerenone initiation (month 1 to 12), excluding the initial acute drop in eGFR to focus on the chronic slope. The y-axis indicates eGFR in mL/min/1.73 m², with tick marks at 0, 15, 30, 45, 60, and 90, which correspond to standard CKD stages. The x-axis represents the months relative to finerenone initiation, where month 0 is the point of treatment initiation. The approximately linear trend of the mean trajectories supports the appropriateness of applying a linear mixed-effects model. eGFR, estimated glomerular filtration rate.

Supplementary Figure S9. Longitudinal changes in eGFR before and after finerenone initiation according to concomitant use of RAS inhibitors.

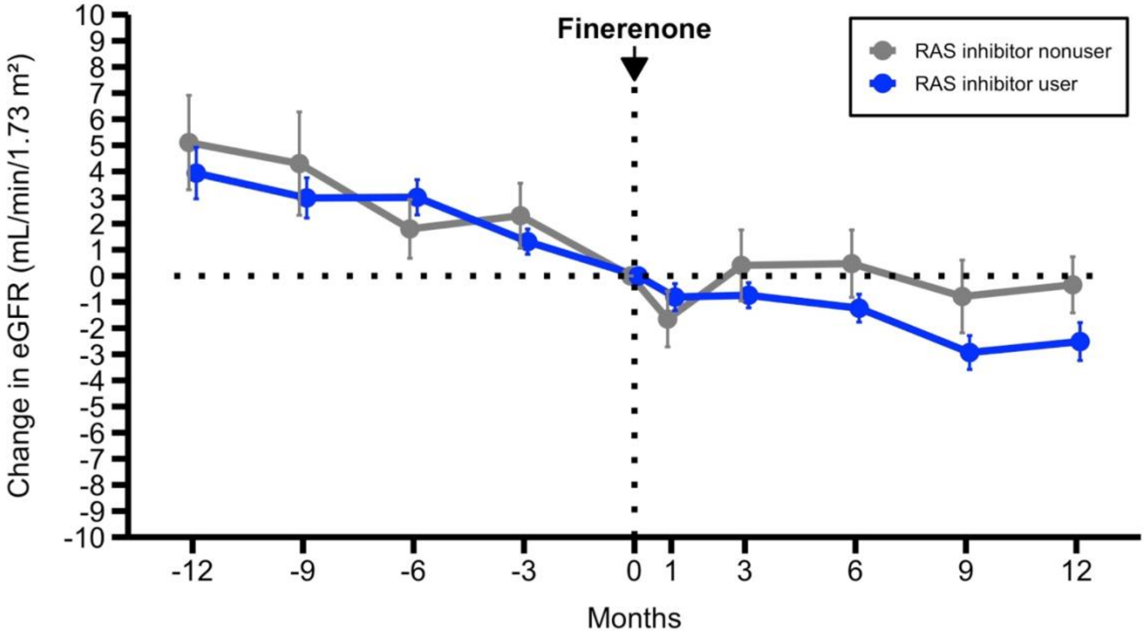

This figure illustrates the longitudinal changes in mean change in eGFR (±SE) before and after finerenone initiation, with the mean eGFR at month 0 centered to 0, according to concomitant use of RAS inhibitors. The blue line represents RAS inhibitor users, while the gray line represents RAS inhibitor nonusers. The eGFR slope during the post-treatment period was calculated from 1 month after finerenone initiation to exclude the acute drop in eGFR observed during the first month. Regardless of RAS inhibitor use, finerenone treatment was associated with an improvement in eGFR slope. Linear mixed-effects models for repeated measures were used to estimate these slopes, with additional details provided in the Supplementary Methods section. eGFR, estimated glomerular filtration rate.

Supplementary Figure S10. Longitudinal changes in eGFR before and after finerenone initiation according to concomitant use of SGLT2 inhibitors.

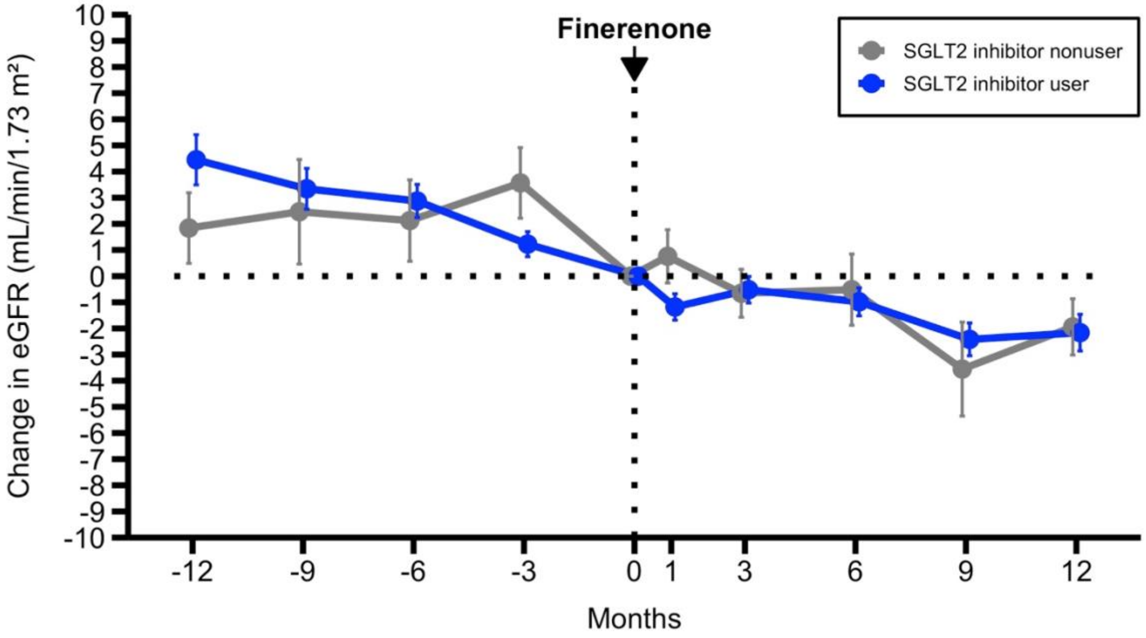

This figure illustrates the longitudinal changes in mean change in eGFR (±SE) before and after finerenone initiation, with the mean eGFR at month 0 centered to 0, according to concomitant use of SGLT2 inhibitors. The blue line represents SGLT2 inhibitor users, while the gray line represents SGLT2 inhibitor nonusers. The eGFR slope during the post-treatment period was calculated from 1 month after finerenone initiation to exclude the acute drop in eGFR observed during the first month. Regardless of SGLT2 inhibitor use, finerenone treatment was associated with an improvement in eGFR slope. Linear mixed-effects models for repeated measures were used to estimate these slopes, with additional details provided in the Supplementary Methods section. eGFR, estimated glomerular filtration rate.

Supplementary Figure S11. Longitudinal changes in eGFR before and after finerenone initiation according to concomitant use of glucagon-like peptide-1 receptor agonists.

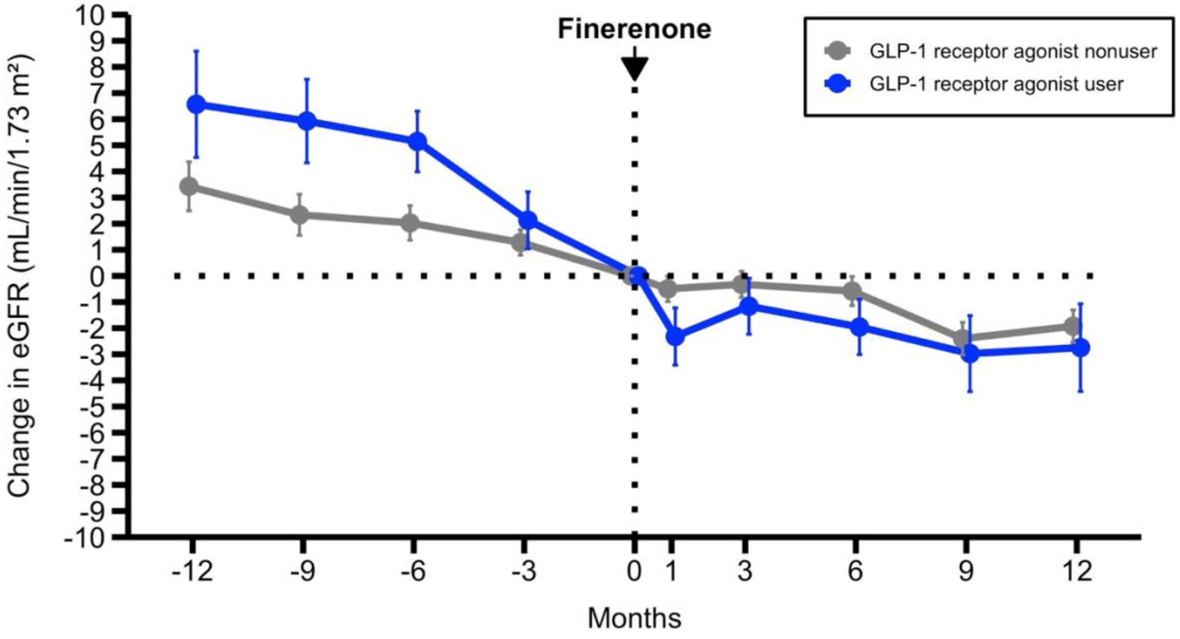

This figure illustrates the longitudinal changes in mean change in eGFR (±SE) before and after finerenone initiation, with the mean eGFR at month 0 centered to 0, according to concomitant use of GLP-1 receptor agonists. The blue line represents GLP-1 receptor agonist users, while the gray line represents GLP-1 receptor agonist nonusers. The eGFR slope during the post-treatment period was calculated from 1 month after finerenone initiation to exclude the acute drop in eGFR observed during the first month. Regardless of GLP-1 receptor agonist use, finerenone treatment was associated with an improvement in eGFR slope. Linear mixed-effects models for repeated measures were used to estimate these slopes, with additional details provided in the Supplementary Methods section. eGFR, estimated glomerular filtration rate; GLP-1 receptor agonist, glucagon-like peptide-1 receptor agonist.

Supplementary Tables

Supplementary Table S1. Baseline characteristics of the study population**.**

**Characteristic**	**Total population (n = 106)**
Age, years, mean (SD)	70.8 (10.4)
Sex, male, n (%)	84 (79.3)
BMI, kg/m^2, mean (SD)	25.2 (4.0)
Duration of diabetes, years, mean (SD)	15.5 (10.1)
CVD history, n (%)	19 (17.9)
Medications, n (%)	
RAS inhibitors	87 (82.1)
SGLT2 inhibitors	94 (88.7)
GLP-1 receptor agonists	27 (25.5)
Statins	69 (65.1)
Diuretics	27 (25.5)
Potassium binders	15 (14.2)
Systolic BP, mmHg, mean (SD)	127.7 (14.6)
Diastolic BP, mmHg, mean (SD)	72.5 (9.8)
HbA1c, %, mean (SD)	6.9 (1.0)
Total cholesterol, mg/dL, median (IQR)	187 (165, 219)
LDL cholesterol, mg/dL, median (IQR)	101 (83, 125)
HDL cholesterol, mg/dL, median (IQR)	50 (42, 67)
Triglycerides, mg/dL, median (IQR)	143 (105, 207)
Serum potassium concentration, mmol/L, mean (SD)	4.3 (0.4)
eGFR, mL/min/1.73 m^2, median (IQR)	38.9 (30.1, 50.9)

eGFR category, n (%)	
≥60	14 (13.2)
45 to <60	24 (22.6)
25 to <45	57 (53.8)
<25	11 (10.4)
ACR, mg/g Crea, median (IQR)	265 (43, 1578)
ACR category, n (%)	
<30	21 (19.8)
30 to 300	33 (31.1)
>300	52 (49.1)

BMI, body mass index; CVD, cardiovascular disease; RAS, renin-angiotensin system; SGLT2, sodium-glucose cotransporter 2; GLP-1, glucagon-like peptide-1; BP, blood pressure; HbA1c, glycated hemoglobin; LDL, low-density lipoprotein; HDL, high-density lipoprotein; eGFR, estimated glomerular filtration rate; ACR, urine albumin-to-creatinine ratio. Data are presented as mean (SD), median (IQR), or n (%).

Supplementary Table S2. Effect of finerenone on eGFR slope changes stratified by baseline eGFR and urine albumin-to-creatinine ratio categories.

	n	Pre-slope	Post-slope	Difference (95% CI)	*P* value
eGFR category					
≥60	14	-2.46 (0.13)	-1.96 (0.19)	0.50 (0.05 to 0.95)	0.032
45 to <60	24	-2.55 (0.24)	-1.76 (0.21)	0.79 (0.16 to 1.42)	0.013
25 to <45	57	-5.25 (0.54)	-1.86 (0.28)	3.39 (2.19 to 4.59)	<0.001
<25	11	-3.22 (0.53)	-1.92 (0.27)	1.30 (0.13 to 2.47)	0.029
ACR category					
<30	21	-2.08 (0.48)	-0.18 (0.38)	1.90 (1.52 to 2.29)	<0.001
30 to 300	33	-4.07 (0.54)	0.38 (0.36)	4.45 (3.46 to 5.44)	<0.001
>300	52	-4.98 (0.50)	-3.78 (0.24)	1.21 (0.11 to 2.30)	<0.001

Pre-slope and post-slope are expressed as mean annualized eGFR slope (mL/min/1.73 m^2/year) with standard error. The difference represents the mean change in eGFR slope between the pre-treatment periods with 95% confidence intervals (CIs). *P* values indicate statistical significane of the difference. eGFR, estimated glomerular filtration rate; ACR, urine albumin-to-creatinine ratio.

Supplementary Table S3. The relationship between acute drop in eGFR and eGFR slope improvement.

**Relationship**	**Correlation coefficient (r)**	***P* value**
Acute drop in eGFR vs. eGFR slope improvement	0.43	<0.001

Pearson's correlation coefficients (r) between acute drop in eGFR and eGFR slope improvement. Correlations with $P < 0.05$ are statistically significant. eGFR, estimated glomerular filtration rate.

STROBE Statement—Checklist of items that should be included in reports of ***cohort studies***

	Item No	Recommendation	Page No
**Title and abstract**	1	(*a*) Indicate the study's design with a commonly used term in the title or the abstract	1
		(*b*) Provide in the abstract an informative and balanced summary of what was done and what was found	N/A
**Introduction**			
Background/rationale	2	Explain the scientific background and rationale for the investigation being reported	3
Objectives	3	State specific objectives, including any prespecified hypotheses	3
**Methods**			
Study design	4	Present key elements of study design early in the paper	Supplementary Methods
Setting	5	Describe the setting, locations, and relevant dates, including periods of recruitment, exposure, follow-up, and data collection	Supplementary Methods
Participants	6	(*a*) Give the eligibility criteria, and the sources and methods of selection of participants. Describe methods of follow-up	Supplementary Methods
		(*b*) For matched studies, give matching criteria and number of exposed and unexposed	N/A
Variables	7	Clearly define all outcomes, exposures, predictors, potential confounders, and effect modifiers. Give diagnostic criteria, if applicable	Supplementary Methods
Data sources/ measurement	8*	For each variable of interest, give sources of data and details of methods of assessment (measurement). Describe comparability of assessment methods if there is more than one group	Supplementary Methods
Bias	9	Describe any efforts to address potential sources of bias	N/A
Study size	10	Explain how the study size was arrived at	Supplementary Figure S1
Quantitative variables	11	Explain how quantitative variables were handled in the analyses. If applicable, describe which groupings were chosen and why	Supplementary Methods
Statistical methods	12	(*a*) Describe all statistical methods, including those used to control for confounding	Supplementary Methods
		(*b*) Describe any methods used to examine subgroups and interactions	Supplementary Methods
		(*c*) Explain how missing data were addressed	N/A
		(*d*) If applicable, explain how loss to follow-up was addressed	N/A
		(*e*) Describe any sensitivity analyses	N/A
**Results**			
Participants	13*	(a) Report numbers of individuals at each stage of study—eg numbers potentially eligible, examined for eligibility, confirmed eligible, included in the study, completing follow-up, and analysed	4, Supplementary Table S1
		(b) Give reasons for non-participation at each stage	Supplementary Figure S1
		(c) Consider use of a flow diagram	Supplementary Figure S1
Descriptive data	14*	(a) Give characteristics of study participants (eg demographic, clinical, social) and information on exposures and potential confounders	4, Supplementary Table S1
		(b) Indicate number of participants with missing data for each variable of interest	N/A
		(c) Summarise follow-up time (eg, average and total amount)	4

Outcome data	15*	Report numbers of outcome events or summary measures over time	4
Main results	16	(*a*) Give unadjusted estimates and, if applicable, confounder-adjusted estimates and their precision (eg, 95% confidence interval). Make clear which confounders were adjusted for and why they were included	4,5 Figure 1, Figure 2
		(*b*) Report category boundaries when continuous variables were categorized	Supplementary Methods, Figure 2, Supplementary Table S2
		(*c*) If relevant, consider translating estimates of relative risk into absolute risk for a meaningful time period	N/A
Other analyses	17	Report other analyses done—eg analyses of subgroups and interactions, and sensitivity analyses	Supplementary Results
**Discussion**			
Key results	18	Summarise key results with reference to study objectives	6-8
Limitations	19	Discuss limitations of the study, taking into account sources of potential bias or imprecision. Discuss both direction and magnitude of any potential bias	8
Interpretation	20	Give a cautious overall interpretation of results considering objectives, limitations, multiplicity of analyses, results from similar studies, and other relevant evidence	8
Generalisability	21	Discuss the generalisability (external validity) of the study results	8
**Other information**			
Funding	22	Give the source of funding and the role of the funders for the present study and, if applicable, for the original study on which the present article is based	9

*Give information separately for exposed and unexposed groups.

Note: An Explanation and Elaboration article discusses each checklist item and gives methodological background and published examples of transparent reporting. The STROBE checklist is best used in conjunction with this article (freely available on the Web sites of PLoS Medicine at http://www.plosmedicine.org/, Annals of Internal Medicine at http://www.annals.org/, and Epidemiology at http://www.epidem.com/). Information on the STROBE Initiative is available at http://www.strobe-statement.org.
